# Long-term neurocognitive function and quality of life after multimodal therapy in adult glioma patients: a prospective long-term follow-up

**DOI:** 10.1007/s11060-023-04419-y

**Published:** 2023-08-30

**Authors:** Milena Pertz, Sabine Schlömer, Clemens Seidel, Bettina Hentschel, Markus Löffler, Gabriele Schackert, Dietmar Krex, Tareq Juratli, Joerg Christian Tonn, Oliver Schnell, Hartmut Vatter, Matthias Simon, Manfred Westphal, Tobias Martens, Michael Sabel, Martin Bendszus, Nils Dörner, Antje Wick, Klaus Fliessbach, Christian Hoppe, Marcel Klingner, Jörg Felsberg, Guido Reifenberger, Dorothee Gramatzki, Michael Weller, Uwe Schlegel, for the German Glioma Network

**Affiliations:** 1 Department of Medical Psychology and Medical Sociology, Ruhr University Bochum, Universitätsstraße 105, 44789 Bochum, Germany; 2 Department of Neurology, University Hospital Knappschaftskrankenhaus, Ruhr University Bochum, Bochum, Germany; 3 Department of Radiation Oncology, University Hospital Leipzig, Leipzig, Germany; 4 Institute for Medical Informatics, Statistics and Epidemiology, University of Leipzig, Leipzig, Germany; 5 Department of Neurosurgery, University Hospital Carl Gustav Carus, Technical University of Dresden, Dresden, Germany; 6 Department of Neurosurgery, University Hospital, Ludwig Maximilians University of Munich, Munich, Germany; 7 Department of Neurosurgery, Medical Center, University of Freiburg, Freiburg, Germany; 8 Department of Neurosurgery, University Hospital Bonn, Bonn, Germany; 9 Department of Neurosurgery, Medical Center Bethel, University Hospital Bielefeld, Bielefeld, Germany; 10 Department of Neurosurgery, University Hospital Hamburg-Eppendorf, Hamburg, Germany; 11 Department of Neurosurgery, Medical Center Asklepios St. Georg, Hamburg, Germany; 12 Department of Neurosurgery, Heinrich Heine University Medical Faculty and University Hospital Düsseldorf, Düsseldorf, Germany; 13 Department of Neuroradiology, Medical Center of Neurology, University Hospital Heidelberg, Heidelberg, Germany; 14 Institute for Diagnostic and Interventional Radiology and Neuroradiology, University Hospital Essen, Essen, Germany; 15 Neurology Clinic and National Centre for Tumour Diseases, University Hospital Heidelberg, Heidelberg, Germany; 16 Clinical Cooperation Unit Neurooncology, German Cancer Research Center (DKFZ), German Cancer Consortium (DKTK), Heidelberg, Germany; 17 Department of Neurodegenerative Diseases and Geriatric Psychiatry, University Hospital Bonn, Bonn, Germany; 18 Department of Epileptology, University Hospital Bonn, Bonn, Germany; 19 Institute of Neuropathology, Heinrich Heine University Medical Faculty and University Hospital Düsseldorf, Düsseldorf, Germany; 20 Department of Neurology, University Hospital and University of Zurich, Zurich, Switzerland; 21 Department of General Neurology, University Hospital Tübingen, Tübingen, Germany; 22 Department of Neurology, Hirslanden Hospital, Zurich, Switzerland

**Keywords:** Glioma, Neurocognition, Quality of life, Radiotherapy, Multimodal tumor-directed treatment

## Abstract

**Purpose:**

Multimodal therapies have significantly improved prognosis in glioma. However, in particular radiotherapy may induce long-term neurotoxicity compromising patients’ neurocognition and quality of life. The present prospective multicenter study aimed to evaluate associations of multimodal treatment with neurocognition with a particular focus on hippocampal irradiation.

**Methods:**

Seventy-one glioma patients (WHO grade 1–4) were serially evaluated with neurocognitive testing and quality of life questionnaires. Prior to (baseline) and following further treatment (median 7.1 years [range 4.6–11.0] after baseline) a standardized computerized neurocognitive test battery (NeuroCog FX) was applied to gauge psychomotor speed and inhibition, verbal short-term memory, working memory, verbal and non-verbal memory as well as verbal fluency. Mean ipsilateral hippocampal radiation dose was determined in a subgroup of 27 patients who received radiotherapy according to radiotherapy plans to evaluate its association with neurocognition.

**Results:**

Between baseline and follow-up mean performance in none of the cognitive domains significantly declined in any treatment modality (radiotherapy, chemotherapy, combined radio-chemotherapy, watchful-waiting), except for selective attention in patients receiving chemotherapy alone. Apart from one subtest (inhibition), mean ipsilateral hippocampal radiation dose > 50 Gy (Dmean) as compared to < 10 Gy showed no associations with long-term cognitive functioning. However, patients with Dmean < 10 Gy showed stable or improved performance in all cognitive domains, while patients with > 50 Gy numerically deteriorated in 4/8 domains.

**Conclusions:**

Multimodal glioma therapy seems to affect neurocognition less than generally assumed. Even patients with unilateral hippocampal irradiation with > 50 Gy showed no profound cognitive decline in this series.

**Supplementary Information:**

The online version contains supplementary material available at 10.1007/s11060-023-04419-y.

## Introduction

Multimodal tumor-specific therapies have significantly improved prognosis of glioma. Nevertheless, apart from the tumor itself therapy-induced long-term neurotoxicity may affect patients’ quality of life (QoL). Surgery, radiotherapy (RT) and chemotherapy (ChT) may negatively impact patients’ cognitive functioning. While the literature on ChT alone is sparse, as many glioma patients receive combined radio-chemotherapy (RChT) [1–3], many investigations focused on the association of RT with cognitive functioning [4–7]. The brain region considered to be particularly vulnerable to RT is the hippocampus, due to the presence of neuronal progenitor cells (NPC) [8–13]. This has translated into therapeutic efforts of hippocampal sparing in brain tumor treatment, mostly in concepts on RT of brain metastases e.g., [14–16], but also in some studies in glioma patients [17, 18]. Data suggest a potential benefit of NPC sparing on neurocognition in glioblastoma [17, 18] but with risk of treatment failure [19].

In a prospective observational study on patients with low grade glioma treated with fractionated stereotactic RT hippocampal dosage was correlated with verbal memory at 18 months follow-up [20]. Biologically equivalent doses of 2 Gy (EQD_2_) fractions (assuming *α*/*β* = 2 Gy) to 40% of the bilateral hippocampus greater than 7.3 Gy were associated with an increased risk of delayed memory impairment [20]. However, in another study on low grade glioma patients treated with proton RT neither a dose–response relationship for the hippocampus nor an overall neurocognitive decline was found at a median of 36 months after treatment [21]. A ground-breaking cross-sectional study on low grade glioma patients who had been irradiated in the 80ies and 90ies of the last century [22, 23] demonstrated that treatment-related cognitive dysfunction is infrequent at a mean of 6 years after diagnosis [22], but affected increasingly more cognitive domains on long-term follow-up (12 years) [23]. The follow-up [23] suggested that around 50% of surviving patients having received RT as primary treatment developed cognitive difficulties even if treated with fractions < 2 Gy. In line with relative stable cognition in the long-term in other studies [6, 24–26], a most recent paper on this population demonstrated that no further cognitive decline was detectable within the next 14 years of follow-up [27].

Most glioma studies with long-term follow-ups were not prospective [22, 23, 27] and prospective studies on hippocampal RT dose [20] did not exceed follow-up testing beyond 18 months. Since our group had not found relevant changes of cognitive performance after multimodal treatment of patients with WHO grade 1–4 gliomas after a median of 16.8 months between post-surgery baseline neuropsychological assessment and follow-up assessment [28], we now present an extended follow-up to assess whether neurocognitive sequelae may occur in the long-term. Neuropsychological and QoL data was prospectively captured in a large multicenter trial (German Glioma Network, GGN) with a follow-up of up to 11 years and with particular focus on the influence of hippocampal RT dosage on long-term neurotoxicity.

## Materials and methods

### Patients

The GGN was funded by the German Cancer Aid (Reference No.: 107940/109459 and 110586) from 2004 to 2012 to establish an interdisciplinary research network of brain tumor treatment in Germany including university hospitals specialized in Neuro-Oncology and reference centers for neuroradiology, neuropathology, molecular diagnostics and biometry. On 01/01/2004 the GGN was initiated and on April 2004 assessment with NeuroCog FX started in participating centers. Overall, 4198 patients were included and treated within the GGN, of whom 280 participated in this project on serial assessment of neurocognitive function between 2005 and 2011. Exclusion criteria for adult glioma patients were presence of aphasia, psychosis or dementia prior to glioma diagnosis and MMSE scores < 20 prior to first neuropsychological assessment (NPA). As patients were recruited 2011 at the latest, tumors had been classified according to the WHO classification of tumors of the central nervous system (CNS) in its versions of 2000 [29] and 2007 [30]. After surgical resection or biopsy, patients had been treated with either conventional external RT, ChT, RChT or watchful-waiting. Participating centers were university hospitals Dresden, Munich (LMU), Bonn, Hamburg, Düsseldorf, Heidelberg and Bochum, Germany. The study was approved by the local ethics committees and the ethics committee of the leading institution (Tübingen, Germany, Registration No.: 353/2003 V) and performed in accordance with the 1964 Declaration of Helsinki and its later amendments. All patients gave written informed consent.

### Neurocognition

NeuroCog FX was used to evaluate various neurocognitive domains whose assessment is recommended for neuro-oncological trials [31, 32]. This computerized test battery compromises eight subtests to gauge domains assumed to be vulnerable [33, 34] to detrimental effects on particular domains of cognition by the tumor itself and by tumor directed therapy, i.e., psychomotor speed and response inhibition (simple, Go-NoGo [selective attention] and interference [response inhibition/flexibility] reaction time; in the following the latter is termed “inhibition”), verbal short-term memory (digit span), working memory (2-back), verbal fluency, verbal and non-verbal (visuospatial) memory. Raw test scores were converted to age-adjusted *z*-scores (mean of 0 and standard deviation, SD of 1) for each subtest. The NPA conducted after surgery (largely within one week [median 7 days, range 4–28]) prior to start of further therapy was considered baseline. Follow-up NPA was intended regularly during clinical follow-up every six months. Since data on every six months was obtainable only in a fraction of patients but long-time follow-up in all, the present long-term follow-up analyzed baseline NPA (timepoint 1, T1) and the latest NPA after baseline assessment (timepoint 2, T2; at maximum 11 years). Cognitive test results of patients with confirmed tumor progression within three months after this NPA were excluded and NPA test results prior to this testing were considered for analyses as time point T2. The effects of any treatment type on any cognitive domain were investigated.

### Quality of life

All patients in this study were scheduled for prospective follow-up with the EORTC-QLQ C30 and BN20 modules every 6 months, but again data were incomplete for some. Therefore, the baseline QoL scores (T1) and the latest QoL assessment at maximum 9 years after baseline assessment (T2) with corresponding neurocognitive data were considered for this study. Since the BN20 module largely represents neurologic dysfunction caused by the tumor itself and to a lesser extent by tumor-specific therapy and since it did not substantially add to the information of the C30 module when focusing on treatment-associated impairment, we only evaluated the functional scales (physical, role, emotional, cognitive and social functioning) and the global score of the C30 module. Scale scores range from 0 (worst level of functioning) to 100 (best level of functioning).

### Radiation and radiation therapy planning

Focal photon RT was applied according to standard of care after CT-planning with 3D conformal RT or intensity modulated RT (IMRT). Delineation of gross tumor volume (GTV), clinical target volume (CTV) and planning target volume (PTV) was performed according to national/international standards for the respective tumor type at that time including fusion of postoperative MRI sequences. Hippocampal sparing was not carried out. The hippocampus was contoured according to recommendations of the RTOG 0933 trial [35]. For the current analysis the hippocampus was delineated post-hoc on available digital treatment plans by an experienced Neuro-Oncologist (C.S.). Hippocampal dosimetry and calculation of mean radiation dose to the ipsilateral hippocampus (Dmean) and near maximum dose to the ipsilateral hippocampus (D1%) was performed. In patients without digital radiation plans Dmean to the ipsilateral hippocampus was extrapolated from isodose lines of paper plans after consensus of C.S. and an experienced medical physicist (M.K.). Further, the volumes of CTVs and PTVs and volume of brain receiving > 30 Gy (V30 Gy) in cc were determined if available from digital plans. V30 Gy was used as a threshold for brain volumes at higher risk for radiation-induced damage [36] as the volume of brain receiving more than 30 Gy is frequently applied to describe normal brain radiation dose exposure, e.g., as one parameter in the EORTC 22033-26033 trial [37] and in other analyses concerning high grade glioma [38, 39].

### Statistical analyses

Concerning clinical and sociodemographic data, *t*-tests for independent samples, one-way analyses of variance (ANOVAs), Fisher’s Exact test and *χ*^2^-test were used. To evaluate changes of cognitive functioning and QoL within any treatment modality (RT, ChT, RChT, watchful-waiting) *t*-tests for dependent samples or repeated-measures ANOVAs were calculated for NeuroCog FX *z*-scores and EORTC-QLQ C30 scores with time of assessment (T1 vs. T2) as within-subject factor and treatment (RT vs. ChT vs. RChT vs. watchful-waiting) as between-subject factor. To control for usage of antiepileptic medication (AED) additional analyses were carried out. For the core analysis concerning radiation dose patients were dichotomized in two groups, those receiving less than Dmean 10 Gy to the ipsilateral hippocampus and those who received a Dmean of more than 50 Gy. Repeated-measures ANOVAs for NeuroCog FX *z*-scores and EORTC-QLQ C30 scores were calculated with time of assessment (T1 vs. T2) as within-subject factor and dose (< 10 Gy vs. > 50 Gy) as between-subject factor. Since group level analyses might obscure individual cognitive deterioration or improvement, the number of patients with a *z*-score ≥ 1.5 below normative mean as a common criteria of clinical relevance (see International Cancer and Cognition Task Force [40]) for each NeuroCog FX subtest was calculated. Nevertheless, this represents an arbitrary definition and while the terminus “clinical relevant” in the manuscript follows this definition it does not imply that the reported changes are not subjectively “meaningful” for individual patients. A difference z-score (T2-T1) was calculated for each NeuroCog FX subtest (i.e., each patient served as own control) and was correlated with radiation data using Pearson correlations. Analyses were performed with SPSS statistics (Version 25) with a level of significance of 0.05.

## Results

### Clinical and sociodemographic characteristics

**Fig. 1:**
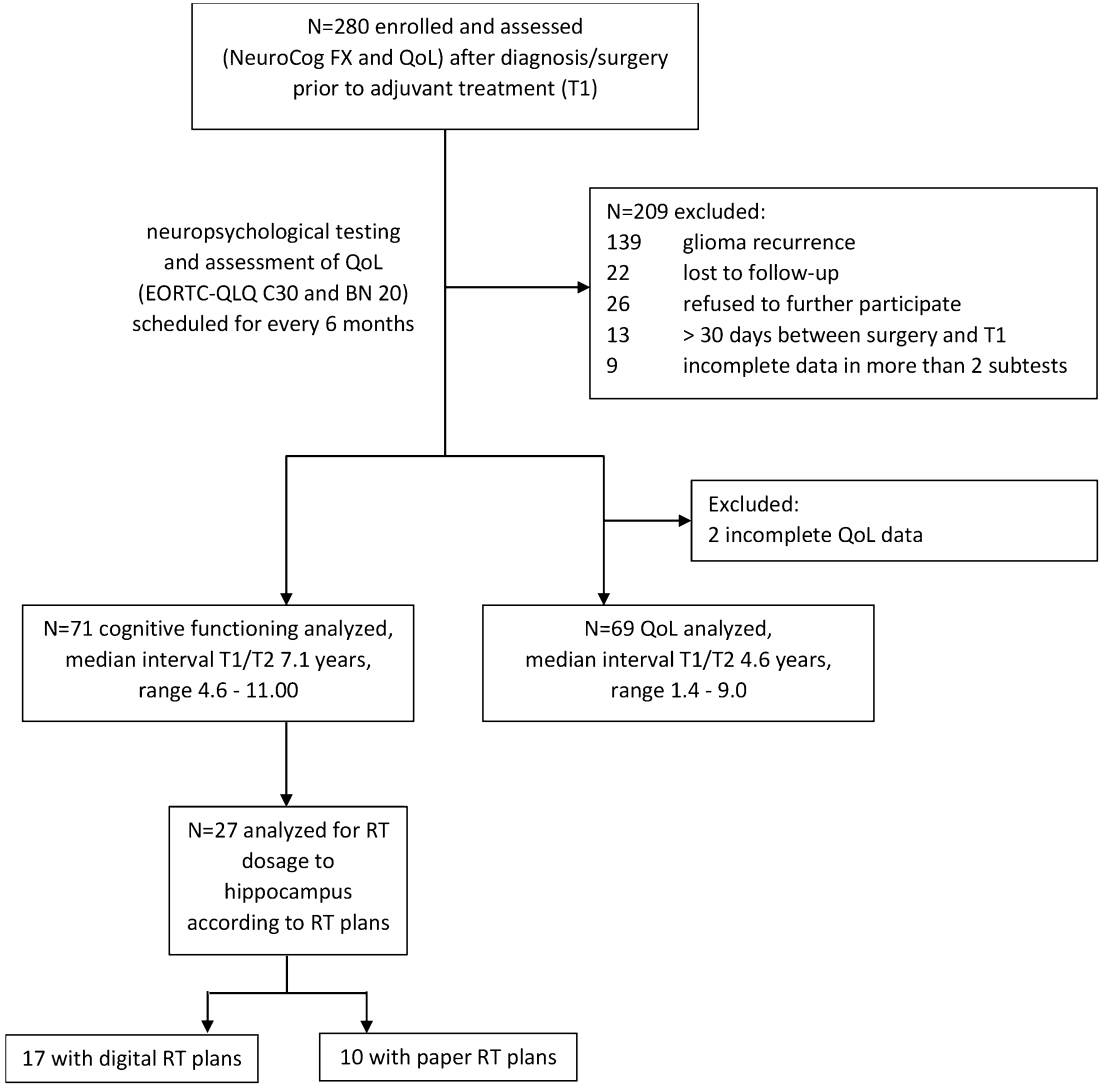
Flow chart of patients included in the present series

Of the 280 patients initially included in this GGN project, 209 patients were excluded from analyses because of missing data in more than two subtests at T1 or T2 (*n* = 9), tumor recurrence before T2 (*n* = 139), an interval > 30 days between surgery and T1 (*n* = 13) or because patients were lost to follow-up (*n* = 22) or refused to participate (*n* = 26). Seventy-one patients with histopathologically proven glioma were prospectively followed in the long-term (Fig. 1 and Table 1).

**Table 1:** Clinical and sociodemographic characteristics of patients, separated for treatment groups and RT dosimetry groups

	Whole sample (*n* = 71)				Sample with RT plans available (*n* = 27)^*^		
	Radiotherapy^a^ (RT)	Combined Radio-Chemotherapy^b^ (RChT)	Chemotherapy^c^ (ChT)	Watchful-waiting	Whole sample^d^	Dmean ipsilateral Hippocampus < 10 Gy	Dmean ipsilateral Hippocampus > 50 Gy
	*n* = 7 (9.9%)	*n* = 29 (40.8%)	*n* = 11 (15.5%)	*n* = 24 (33.8%)	*n* = 27	*n* = 8 (29.6%)	*n* = 12 (44.4%)
Median age in years (range) at surgery	39 (30–58)	36 (21–53)	42 (27–52)	34 (17–50)	37.0 (21–58)	35.0 (21–44)	35.5 (25–47)
Sex, n, female : male	3:4 (43%:57%)	13:16 (45%:55%)	9:2 (82%:18%)	13:11 (54%:46%)	14:13 (52%:48%)	5:3 (63%:37%)	6:6 (50%:50%)
Education in years							
Mean (SD)	12.1 (1.9)	11.8 (1.6)	12.1 (1.6)	11.6 (1.4)	11.8 (1.7)	12.3 (1.5)	11.9 (2.0)
Surgery, n							
Gross total resection ^e^	3	8	4	17	9	2	4
Subtotal resection ^e^	0	6	2	5	5	3	1
Partial resection ^e^	1	11	1	1	7	–	4
Biopsy (open vs. stereotactic)	3 (stereotactic)	4 (1 vs. 3)	4 (1 vs. 3)	1 (stereotactic)	6 (1 vs. 5)	3 (1 vs. 2)	3 (stereotactic)
Tumor histology, according to the WHO classification 2000 and 2007, n							
Astrocytoma	1	9	2	6	8	2	4
Anaplastic astrocytoma	4	9	2	2	12	3	5
Oligodendroglioma	–	1	–	1	1	1	–
Anaplastic oligodendroglioma	–	1	2	–	1	–	1
Oligoastrocytoma	1	–	3	5	1	–	–
Anaplastic oligoastrocytoma	–	4	2	1	1	1	–
Glioblastoma	–	5	–	–	3	1	2
Other	1 (ganglioglioma)	–	–	9 (7 pilocytic astrocytoma, 1 subependymoma, 1 ganglioglioma)	–	–	–
WHO grade, according to the WHO classification 2000 and 2007, n							
Grade 1	1	–	–	8	–	–	–
Grade 2	2	10	5	13	10	3	4
Grade 3	4	14	6	3	14	4	6
Grade 4	–	5	–	–	3	1	2
Lateralization of tumor, n							
Left	5	18	2	9	18	3	11
Right	2	10	9	14	9	5	1
Bilateral	–	1	–	–	–	–	–
Crossing midline	–	–	–	1	–	–	–
Localization of tumor, n							
Frontal	2	9	9	9	9	7	1
Temporal	4	7	–	3	11	–	8
Parietal	–	1	2	1	5	–	2
Fronto-temporal	–	6	–	1	2	1	1
Temporo-parietal	–	2	–	1	–	–	–
Other	1	4	–	9	–	–	–
AED at T1, n	2	20	7	10	15	5	5
AED at T2, n	3	10	5	8	7	1	3
Time interval NPA T1 – T2 in mo							
Mean (SD)	79.7 (16.8)	93.3 (19.3)	85.8 (24.0)	85.5 (20.1)	94.2 (20.4)	92.7 (16.2)	88.6 (21.3)
Median (range)	78.3 (57.8–109.0)	88.4 (61.6–129.6)	83.9 (61.2–131.6)	82.6 (55.3–128.7)	88.4 (57.8–129.6)	88.6 (72.3–114.4)	84.4 (57.8–127.2)
Time interval QoL T1 – T2 in mo							
Mean (SD)	45.4 (28.9)	61.5 (20.1)	50.5 (21.1)	54.9 (26.6)	61.2 (22.7)	63.4 (20.2)	55.3 (21.6)
Median (range)	33.0 (24.0–107.0)	68.0 (25.0–97.0)	47.0 (25.0–82.9)	55.5 (17.0–108.0)	61.0 (24.0–107.0)	73.0 (29.0–88.0)	52.5 (24.0–87.9)
Time interval surgery – NPA T1 in d							
Mean (SD)	8.7 (4.6)	9.2 (5.0)	9.8 (9.0)	7.4 (1.4)	10.3 (5.5)	10.4 (5.9)	7.0 (1.6)
Median (range)	7 (5–16)	7 (4–20)	7 (5–28)	8 (4–10)	8 (4–20)	11 (4–19)	7 (5–9)
Time interval surgery – QoL T1 in d							
Mean (SD)	9.0 (4.7)	10.0 (9.6)	8.0 (5.1)	31.0 (37.5)	11.3 (11.1)	10.2 (5.8)	6.7 (.6)
Median (range)	7 (5–19)	7 (4–42)	7 (4–18)	7 (4–97)	7 (4–42)	10 (4–19)	7 (6–7)
Time interval NPA T1 – adjuvant therapy onset in d							
Mean (SD)	24.3 (11.2)	31.7 (14.0)	23.2 (25.9)	–	31.3 (15.8)	34.2 (16.4)	33.5 (13.6)
Median (range)	18 (6–34)	34 (5–51)	15 (5–73)	–	40 (5–48)	40 (5–44)	33 (20–48)
Time interval QoL T1 – adjuvant therapy onset in d							
Mean (SD)	33.8 (19.3)	32.3 (15.9)	22.1 (22.3)	–	31.3 (17.7)	34.2 (15.8)	37.0 (15.7)
Median (range)	35 (6–49)	37 (6–57)	17.5 (5–73)	–	40 (6–57)	40 (6–43)	44 (19–48)

*SD* standard deviation, *mo* months, *d* days, *AED* anti-epileptic drug, *T1* baseline assessment, *T2* follow-up assessment; *NPA* neuropsychological assessment, *QoL* Quality of life

^a^All patients received external focal RT

^b^Adjuvant: 20.7% temozolomide; concomitant: 37.9% temozolomide; 41.4% PCV or nitrosourea

^c^Adjuvant: 63.6% temozolomide, 36.4% PCV or nitrosourea

^d^Six (22.2%) patients received external focal RT alone and 21 (77.8%) were treated with radio-chemotherapy (adjuvant: 23.8% temozolomide; concomitant: 28.6% temozolomide; 47.6% PCV or nitrosourea)

^e^Extent of resection was defined according to magnetic resonance (MR) or computer tomography (CT) imaging within 21 days post-surgery. Post-surgical residual tumor volume was compared to tumor volume prior to surgery. Gross total resection was defined as no visible residual tumor, subtotal resection as 50–99% excision of tumor volume and partial resection as < 50% excision of tumor volume

^*^For *n* = 6 patients hippocampal dosage was between 10 and 50 Gy; for *n* = 1 patient estimation of hippocampal dosage was not possible; data of these patients were not included in one of predefined dichotomized groups

The median follow-up was 85.2 months (range 55.3–131.6) after baseline assessment for neurocognition and 55.0 months (range 17.0–108.0) for QoL. Patients having received RT were treated with fraction doses of 1.8–2.0 Gy with a median total dose of 59.4 Gy (range 39.6–60.0). More patients with WHO grade 3 and 4 gliomas were treated with RChT and more patients with WHO grade 1 and 2 gliomas received watchful-waiting (*p* = 0.001). Patients who have been treated with RChT harbored more often left-sided tumors, whereas patients who received ChT or watchful-waiting harbored more often right-sided tumors (*p* = 0.038). No further differences between treatment groups concerning clinical and sociodemographic characteristics were noted (Online Resource Table S1).

Of the 71 included, 27 had RT plans available (Table 1). The median follow-up of these was 88.4 months (range 57.8–129.6) after baseline assessment for neurocognition and 61.0 months (range 24.0–107.0) after baseline assessment for QoL. The median total dose was 59.4 Gy (range 50.4–60.0), fraction doses were 1.8–2.0 Gy. In 6 of these patients (22.2%) treatment concept and total dose included a local boost RT (median total boost dose 14.4 Gy [range 7.2–20.0]) in fractions of 1.8–2.0 Gy. Two patients were irradiated with IMRT, the remaining patients were irradiated with 3D conformal RT. The median PTV was 230.0 cc (range 101.0–566.0), median CTV was 79 cc (range 36.0–214.0). Dmean to the ipsilateral hippocampus was 43.7 Gy (range 2.2–59.6) for patients with digital RT plans (*n* = 6 < 10 Gy; *n* = 8 > 50 Gy; *n* = 2 10–50 Gy; *n* = 1 not estimated since hippocampus was fully resected before RT). For the remaining Dmean to the ipsilateral hippocampus was dichotomized into < 10 Gy (*n* = 2) and > 50 Gy (*n* = 4) extrapolated from paper plans. In 4 with paper plans Dmean hippocampus was between 10 and 50 Gy. It is of note that patients with Dmean between 10 and 50 Gy were not included in the core analysis of dichotomized groups. From inspection of planning imaging in 7 patients hippocampal structures had been resected before RT (6 partial resection, 1 complete resection). With the exception of a statistically significant group difference in tumor lateralization (*p* = 0.013) and in localization, i.e., frontal vs. non-frontal tumors (*p* = 0.001) no differences between groups concerning clinical and sociodemographic characteristics were noted (Online Resource Table S1).

### Long-term follow-up of neurocognition and QoL in the whole cohort

**Fig. 2:**
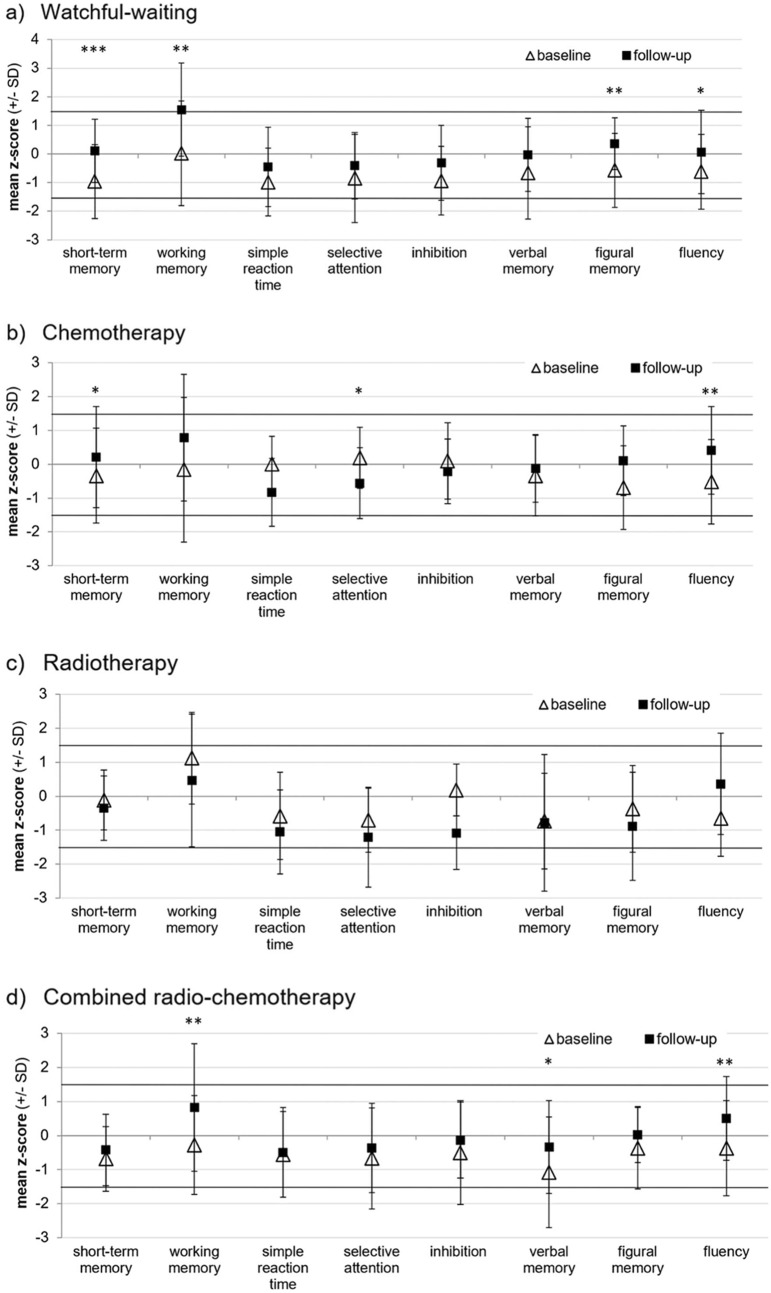
Mean cognitive performance (in *z*-scores) and standard deviations (error bars) in NeuroCog FX subtests at baseline and follow-up (median 7.1 years [range 4.6–11.0] after baseline), separated for treatment groups. **a** Watchful-waiting (*n* = 24), **b** Chemotherapy (*n* = 11), **c** Radiotherapy (*n* = 7), **d** Combined radio-chemotherapy (*n* = 29). Statistically significant changes in cognitive performance between baseline and follow-up are indicated by asterisks (*p < .05, **p < .01, ***p < .001)

Mean performance of all treatment groups at T1 and T2 indicated no clinically relevant impairment (i.e., performance was within the range of 1.5 SD around normative mean; Fig. 2).

RChT and watchful-waiting patients showed numerical and sometimes even statistically significant improvements in all cognitive domains (Fig. 2 and Online Resource Table S2). For RT patients no statistically significant changes (yet numerical improvements or deteriorations) were seen in the long-term. ChT patients significantly improved in short-term memory (*p* = 0.046) and fluency (*p* = 0.001). Although ChT patients significantly deteriorated in one single domain (selective attention, *p* = 0.017), performance was still within normative range. ANOVAs revealed differences with respect to the extent of change in neurocognitive performance between treatment groups (interaction effects) in *short-term memory*, *inhibition* and *figural memory,* due to differences between watchful-waiting and RT patients (Online Resource Table S2). The number of individual patients in different treatment groups with a clinically relevant impairment of cognitive performance (≥ 1.5 SD below normative mean) is shown in Online Resource Table S3.

As AED may affect cognitive performance and their omission may substantially improve neurocognition, exploratory analyses were conducted to control for an effect of AED discontinuation between T1 and T2 on cognitive performance. Seven patients received anticonvulsants at T1 but not at T2. Individual analyses showed that a confounding effect of these 7 on the extent of neurocognitive improvement at T2 in the whole cohort is highly unlikely (Online Resource Table S4).

Patients who received RChT showed a statistically significant improvement in the EORTC-QLQ C30 scale social functioning (*p* = 0.049) and Global Health Status (*p* = 0.025) in the long-term. Patients undergoing watchful-waiting presented an improvement in Global Health Status (*p* = 0.044). No further significant changes occurred concerning other C30 functional scales or Global Health Score in these treatment groups. Concerning the other treatment groups (ChT and RT) no significant changes on QoL occurred (Online Resource Figure S5).

### Association of hippocampal RT dose with neurocognition and QoL

**Fig. 3:**
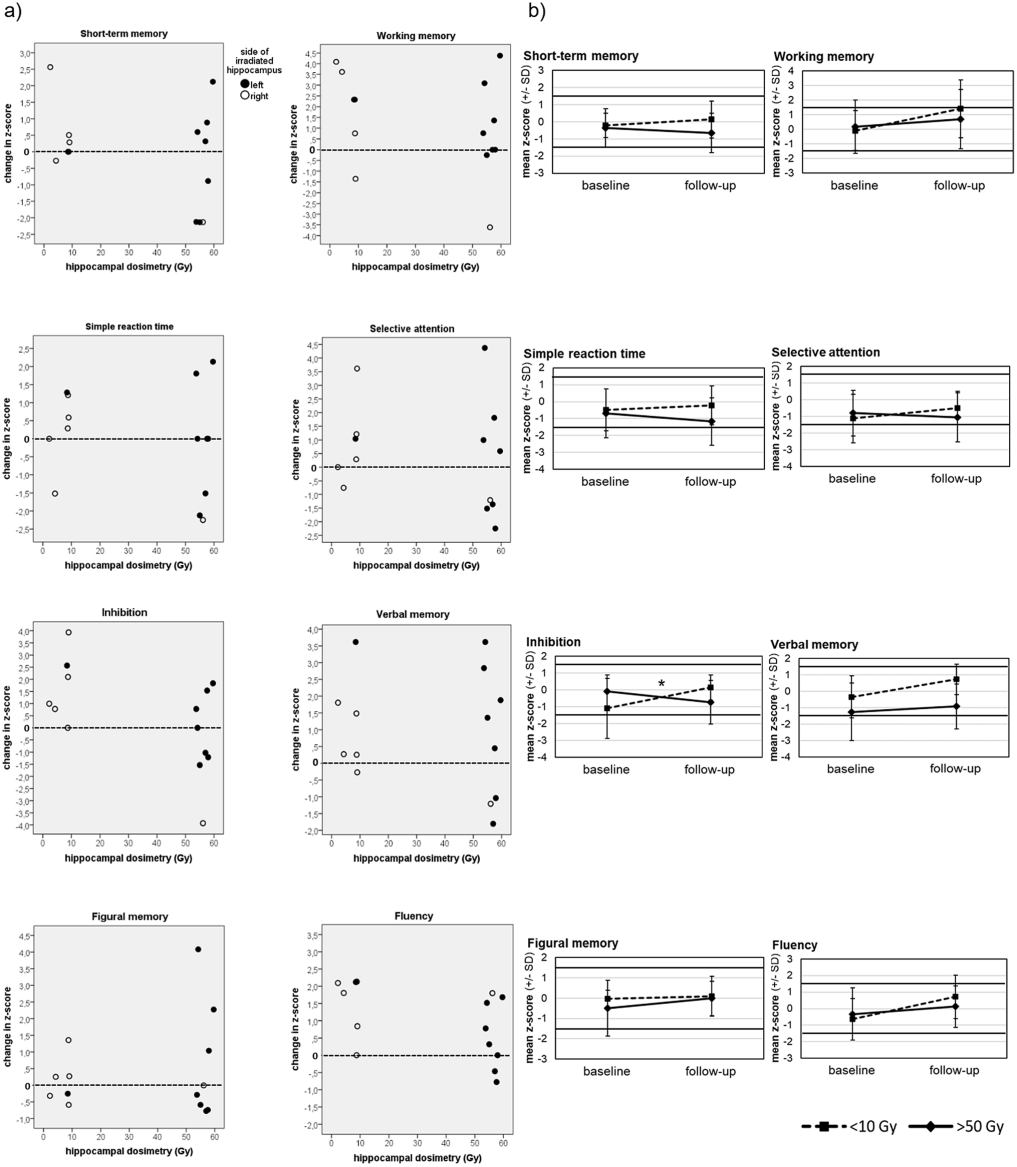
Cognitive performance in relation to hippocampal dosimetry. **a** Change in cognitive performance according to mean radiation dose on ipsilateral hippocampus on individual patient level. *Z*-scores > 0 indicate an improvement of cognitive performance, *z*-scores < 0 indicate a deterioration of cognitive performance. Individual data only refer to digital RT plans with exact hippocampal dosage in Gy available for patient groups with Dmean at ipsilateral Hippocampus < 10 Gy vs. > 50 Gy **b** Change of cognitive functioning in the long-term on group level. Graphs indicate mean cognitive performance (*z*-scores) with standard deviations represented by error bars in NeuroCog FX subtests at baseline (i.e., after surgery) and at follow-up (median 7.1 years [range 4.6–11.0] after baseline), separated for patients with Dmean at ipsilateral Hippocampus < 10 Gy (*n* = 8) and patients with Dmean at ipsilateral Hippocampus > 50 Gy (*n* = 12). The asterisk indicates a statistically significant interaction effect of timepoint and group (*p < .05)

Change in cognitive performance according to Dmean ipsilateral hippocampus on an individual patient level is presented in Fig. 3a.

Repeated-measures ANOVAs revealed a significant interaction for inhibition (*p* = 0.020) only, i.e., a difference in long-term cognitive change between groups: patients with mean ipsilateral hippocampal dose < 10 Gy improved (*z*-score -1.09 at T1; *z*-score 0.14 at T2), whereas patients with mean ipsilateral hippocampal dose > 50 Gy deteriorated (*z*-score -0.10 at T1; z-score -0.73 at T2) (Fig. 3b and Online Resource Table S6). The number of individual patients with a clinically relevant impairment of cognitive performance ≥ 1.5 SD below normative mean at T1 and T2 is shown in Online Resource Table S3. Group mean scores, irrespective of radiation dose on ipsilateral hippocampus, indicate no clinically relevant cognitive impairment (no z-score was ≥ 1.5 SD below normative mean) in any subtest at T2 (Fig. 3b).

Repeated-measures ANOVAs revealed no significant interaction effects between timepoint and group for C30 functional scales and Global Health Status in the long-term, i.e., no significant association of hippocampal RT and QoL (Online Resource Fig. S7 and Table S8).

The changes in *z*-scores between T1 and T2 were not correlated with PTV, CTV, V30 Gy, Dmean or D1% in any cognitive domain (all *p*s > 0.054; Table 2 and Online Resource Fig. S9).

**Table 2:** Pearson correlation coefficients of correlation between planning target volume, clinical target volume, V30 Gy, mean dose in ipsilateral hippocampus (Dmean), near maximum dose in ipsilateral hippocampus (D_1%_) and change in *z*-scores (T2–T1) in NeuroCog FX subtests

	Planning target volume	Clinical target volume	V30 Gy	Dmean ipsilateral hippocampus	D_1%_ ipsilateral hippocampus
Short-term memory					
Pearson’s r	− .132	− .314	− .040	− .342	− .381
* p*-value	.519	.295	.879	.195	.146
* N*	26	13	17	16	16
Working memory					
Pearson’s r	− .060	− .236	.013	− .329	− .314
* p*-value	.776	.438	.962	.232	.255
* N*	25	13	16	15	15
Simple reaction time					
Pearson’s r	− .077	.353	.357	− .075	.020
* p*-value	.709	.236	.159	.784	.940
* N*	26	13	17	16	16
Selective attention					
Pearson’s r	− .158	− .075	− .101	− .168	− .140
* p*-value	.440	.807	.700	.534	.606
* N*	26	13	17	16	16
Inhibition					
Pearson’s r	− .199	.055	.035	− .491	− .455
* p*-value	.329	.858	.894	.054	.077
* N*	26	13	17	16	16
Verbal memory					
Pearson’s r	− .080	− .210	− .064	− .166	− .100
* p*-value	.698	.491	.808	.539	.712
* N*	26	13	17	16	16
Left tumor lateralization					
Pearson’s r	.028	− .210	.013	− .498	− ,407
* p*-value	.914	.587	.975	.209	.316
* N*	17	9	9	8	8
Right tumor lateralization					
Pearson’s r	− .694	− .430	− .678	− .701	− .772
* p*-value	.038*	.570	.065	.053	.025*
* N*	9	4	8	8	8
Figural memory					
Pearson’s r	.117	− .281	− .005	.158	.107
* p*-value	.569	.352	.985	.558	.694
* N*	26	13	17	16	16
Left tumor lateralization					
Pearson’s r	.374	− .325	.089	.193	.297
* p*-value	.139	.394	.819	.647	.475
* N*	17	9	9	8	8
Right tumor lateralization					
Pearson’s r	− .614	− .470	− .516	− .252	− .414
* p*-value	.079	.530	.190	.548	.308
* N*	9	4	8	8	8
Fluency					
Pearson’s r	.106	.047	− .026	− .429	− .294
* p*-value	.607	.879	.920	.097	.269
* N*	26	13	17	16	16

*r* correlation coefficient, *N* number of patients, *Gy* gray

As verbal memory is associated with left hippocampal integrity and figural memory with right hippocampal function, we additionally separately calculated correlations between change in verbal/figural memory and irradiation of left/right hippocampus. Only for verbal memory, changes in z-score between T1 and T2 were correlated with irradiation of right hippocampus (Table 2).

## Discussion

Long-term neurocognitive and psychosocial sequalae of multimodal tumor-directed treatment are increasingly important in glioma patients. It appears pivotal to prospectively assess the genuine neurotoxicity of tumor-directed treatment.

In our series we did not find profound changes in neurocognitive functioning or QoL on long-term in any of the treatment groups. Although cognitive performance in several patients was numerically below baseline, the mean neurocognitive scores showed no clinically relevant impairment [40] in all treatment modalities. Furthermore, in the current patient sample no profound QoL decreases were present, instead in most cases QoL was increased in the follow-up. Hence, tentatively speaking tumor control might be associated with improved QoL in the patients irrespective of the therapy chosen. However, we cannot exclude the possibility of subjectively meaningful impairments of individual patients. Nevertheless, in accordance with findings in the literature [6, 24–26, 41] patients generally retained their cognitive functioning after treatment. As expected, patients receiving a mean radiation dose of < 10 Gy to the ipsilateral hippocampus showed stable or improved performance in all cognitive domains on group level, while patients with > 50 Gy numerically deteriorated in 4/8 domains. However, no profound impairment was present even in patients treated with > 50 Gy. The stable neurocognitive performance in the long-term might be due to a biphasic pattern of cognitive functioning following RT, as shown in a pioneering study on cognitive decline early after RT with a slope of improvement 2 and 3 years after [42]. Furthermore, we did not find an association of neurocognition and unilateral hippocampal RT dosage across all patients in this series. Only in right-hemispheric patients PTV and D1% of ipsilateral hippocampus were associated with verbal memory which is unexpected since theoretical models suggest an association of left-hemispheric RT with verbal memory. Although the sample size of patients whose radiation plans were available is small and may not be representative the present results suggest that RT to ipsilateral hippocampus in the population analyzed here is only modestly neurotoxic. At least, a group of patients may tolerate therapy in long-term good health, preserved QoL [43] and without gross neurocognitive impairment.

When descriptively evaluated a positive potential effect on neurocognition might be present in the watchful-waiting and RChT groups. Although these marginal effects must be interpreted with caution, one might assume that in terms of tumor control combination RChT is superior to either modality administered alone [44]. Therefore, a positive effect on neurocognition after combined therapy may reflect more efficient tumor control in the RChT group. In the watchful-waiting group we analyzed neurocognition during a long course without detectable tumor progression in this subgroup (median: 85.2 months; range 55.3–131.6). Hence, these results might possibly reflect the avoidance of potential neurotoxic treatment in a positive selection of patients with an exceptionally benign clinical course.

Our results must be interpreted with caution. NeuroCog FX is possibly not sensitive enough to detect subtle neurocognitive impairment. Nevertheless, it reportedly was sensitive in other brain tumor trials [45, 46] and was compared to established neuropsychological testing [34]. As patients with radiation plans were recruited predominantly in one center, we further cannot exclude the possibility of a confounding effect of study site. However, data collection of the whole cohort was nearly equally distributed across centers and did not reveal significant impairments due to multimodal tumor-directed treatment. Of the 280 patients initially included, we prospectively followed only 71 patients (Fig. 1). Hence, we cannot exclude the possibility that we report on a positive selection. Patients who refuse to participate may be in a clinically worse (neurocognitive) condition. Nevertheless, only 9.3% of our sample refused to participate and this limitation might be inherent for prospective studies spanning multiple years. Additionally, due to limited number of patients with RT plans available, we were unable to perform subgroup analyses on different tumor histology, presurgical tumor volume, tumor locations/hemispheres, varying radiation volumes and exact RT doses to the hippocampus. Furthermore in contrast to other research [47], besides formal cognitive test results the present study did not assess patients’ performance status over time, nor their professional and social activities such as resuming employment or hobbies, taking part in further education, maintaining social relationships, living independently or suffering from psychiatric illness, all aspects that define the human condition beyond formal cognitive tests. Finally, O^6^-methylguanine DNA methyltransferase (*MGMT*) promoter methylation was not uniformly assessed and isocitrate dehydrogenase (IDH) mutation status remains unknown for most patients, given the diagnostic classification based on WHO classification of 2000 [29] and 2007 [30]. This is attributable to the time period in which the study was conducted, and it is unlikely that having access to this information would provide further insight into the observations reported here, given that tumor genotype is not commonly associated with neurocognitive outcomes.

Besides these limitations the present study represents the first to serially follow a large number of adult glioma patients for up to 11 years by serial neuropsychological testing, QoL assessment and analyses of hippocampal RT dosage in association with neurocognition in a subgroup of patients with RT plans available. Although we might present the results of a positive selection of patients, gross impairment of neurocognition or QoL appears unlikely in adult glioma patients undergoing unilateral hippocampal RT in this very cohort. Although understanding of mechanisms of how RT ± ChT may or may not result in neurocognitive dysfunction in the long-term remains a matter of further investigation, the present study provides a hint that at least some patients tolerate these therapies without gross neurocognitive and QoL decline.

### Supplementary Information

Below is the link to the electronic supplementary material.

Supplementary file1 (PDF 115 KB)

Supplementary file2 (PDF 59 KB)

Supplementary file3 (PDF 89 KB)

Supplementary file4 (PDF 36 KB)

Supplementary file5 (PDF 292 KB)

Supplementary file6 (PDF 24 KB)

Supplementary file7 (PDF 265 KB)

Supplementary file8 (PDF 21 KB)

Supplementary file9 (PDF 353 KB)

## Data Availability

The datasets generated and/or analysed during the current study are available from Sabine Schlömer (sabine.schloemer@kk-bochum.de) on reasonable request.
