# Supplementary material for: Long-term neurocognitive function and quality of life after multimodal therapy in adult glioma patients: a prospective long-term follow-up
Source: J Neurooncol. 2023 Aug 30;164(2):353–66. doi: 10.1007/s11060-023-04419-y (PMC10522752; doi:10.1007/s11060-023-04419-y)
Supplement: Supplementary file 1 — Supplementary file1 (PDF 115 KB) [file 11060_2023_4419_MOESM1_ESM.pdf]

Online Resource for the manuscript entitled: Long-term neurocognitive function and quality of life after multimodal therapy in adult glioma patients: A prospective long-term follow-up

Milena Pertz, Sabine Schlömer, Clemens Seidel, Bettina Hentschel, Markus Löffler, Gabriele Schackert, Dietmar Krex, Tareq Juratli, Joerg Christian Tonn, Oliver Schnell, Hartmut Vatter, Matthias Simon, Manfred Westphal, Tobias Martens, Michael Sabel, Martin Bendszus, Nils Dörner, Antje Wick, Klaus Fliessbach, Christian Hoppe, Marcel Klingner, Jörg Felsberg, Guido Reifenberger, Dorothee Gramatzki, Michael Weller, Uwe Schlegel for the German Glioma Network

Corresponding author: Milena Pertz

E-Mail address: milena.pertz@rub.de

Department of Medical Psychology and Medical Sociology, Ruhr University Bochum

Universitätsstraße 105, D-44789 Bochum, Germany

Journal name: Journal of Neuro-Oncology

Online Resource Table S1 Comparison of frequencies and means of clinical and sociodemographic characteristics, separated for treatment groups (RT [n=7] vs. ChT [n=11] vs. RChT [n=29] vs. watchful-waiting [n=24]) and RT dosimetry groups (Dmean ipsilateral Hippocampus < 10 Gy [n=8] vs. > 50 Gy [n=12])

	**Whole sample (n=71)**	**Sample with RT plans available (n=27)**[#]
	**Comparison of treatment groups**<br>**RT vs. ChT vs. RChT vs. watchful-waiting**	**Comparison of RT dosage groups**<br>**< 10 Gy vs. > 50 Gy**
Median age in years at surgery	$F(3,70) = 2.563$; $p = .062$	$F(1,19) = .096$; $p = .760$
Sex, n	χ^2 (3) = 4.746; $p = .191$	*Exact* χ^2; $p = .670$
Education in years	$F(3,69) = .407$; $p = .749$	$F(1,19) = .164$; $p = .690$
Surgery, n (gross total, subtotal, partial resection, open, stereotactic biopsy)	χ^2 (12) = 18.381; $p = .105$	χ^2 (4) = 3.263; $p = .515$
WHO grade, according to the WHO classification 2000 and 2007, n	χ^2 (9) = 28.338; $p = .001$**	χ^2 (2) = .079; $p = .961$
Lateralization of tumor (left vs. right), n	χ^2 (3) = 8.442; $p = .038$*	χ^2 (1) = 6.115; $p = .013$*
Localization of tumor, n		
Frontal vs. non-frontal	χ^2 (3) = 6.712; $p = .082$	*Exact* χ^2; $p = .001$**
Temporal vs. non-temporal	χ^2 (3) = 5.439; $p = .142$	*Exact* χ^2; $p = .055$
AED at T1, n	χ^2 (3) = 6.314; $p = .097$	*Exact* χ^2; $p = .650$
AED at T2, n	χ^2 (3) = .524; $p = .914$	*Exact* χ^2; $p = .635$
Time interval NPA T1 – T2 in mo	$F(3,70) = 1.244$; $p = .301$	$F(1,19) = .206$; $p = .655$
Time interval QoL T1 – T2 in mo	$F(3,70) = 1.174$; $p = .326$	$F(1,19) = .705$; $p = .412$
Time interval surgery – NPA T1 in d	$F(3,70) = .769$; $p = .471$	$F(1,19) = 1.238$; $p = .303$
Time interval surgery – QoL T1 in d	$F(3,70) = 3.2$; $p = .054$	$F(1,19) = 1.022$; $p = .351$
Time interval NPA T1 – adjuvant therapy onset in d	$F(2,70) = .980$; $p = .335$	$F(1,19) = .005$; $p = .947$
Time interval QoL T1 – adjuvant therapy onset in d	$F(2,68) = 1.67$; $p = .210$	$F(1,19) = .059$; $p = .816$

Note: *d* days, *mo* months, *AED* anti-epileptic drug, *NPA* neuropsychological assessment, *QoL* Quality of life
[#] for n=6 patients hippocampal dosage was between 10 Gy and 50 Gy; for n=1 patient estimation of hippocampal dosage was not possible; data of these patients were not included in one of predefined dichotomized groups. Asterisks indicate statistically significant differences between groups, * $p < .05$, ** $p < .01$
