# Supplementary material for: Long-term neurocognitive function and quality of life after multimodal therapy in adult glioma patients: a prospective long-term follow-up
Source: J Neurooncol. 2023 Aug 30;164(2):353–66. doi: 10.1007/s11060-023-04419-y (PMC10522752; doi:10.1007/s11060-023-04419-y)
Supplement: Supplementary file 2 — Supplementary file2 (PDF 59 KB) [file 11060_2023_4419_MOESM2_ESM.pdf]

Online Resource for the manuscript entitled: Long-term neurocognitive function and quality of life after multimodal therapy in adult glioma patients: A prospective long-term follow-up

Milena Pertz, Sabine Schlömer, Clemens Seidel, Bettina Hentschel, Markus Löffler, Gabriele Schackert, Dietmar Krex, Tareq Juratli, Joerg Christian Tonn, Oliver Schnell, Hartmut Vatter, Matthias Simon, Manfred Westphal, Tobias Martens, Michael Sabel, Martin Bendszus, Nils Dörner, Antje Wick, Klaus Fliessbach, Christian Hoppe, Marcel Klingner, Jörg Felsberg, Guido Reifenberger, Dorothee Gramatzki, Michael Weller, Uwe Schlegel for the German Glioma Network

Corresponding author: Milena Pertz

E-Mail address: milena.pertz@rub.de

Department of Medical Psychology and Medical Sociology, Ruhr University Bochum

Universitätsstraße 105, D-44789 Bochum, Germany

Journal name: Journal of Neuro-Oncology

Online Resource Table S2 Neurocognitive change within and between treatment groups. T-test statistics for mean extent of cognitive change (i.e. mean difference [in z-scores] between baseline [T1] and follow-up [T2] neuropsychological assessment; a z-score > 0 indicates an improvement of cognitive performance, a z-score < 0 indicates a deterioration of cognitive performance), separated for patients with radiotherapy (RT), chemotherapy (ChT), combined radio-chemotherapy (RChT) and patients followed by watchful-waiting strategy.

	**RT (n = 7)**			**ChT (n = 11)**			**RChT (n = 29)**			**Watchful-waiting (n = 24)**			**ANOVA[#]**		
	Mean change between T1 and T2 (SD) [in z-scores]	t-value (df)	p-value	Mean change between T1 and T2 (SD) [in z-scores]	t-value (df)	p-value	Mean change between T1 and T2 (SD) [in z-scores]	t-value (df)	p-value	Mean change between T1 and T2 (SD) [in z-scores]	t-value (df)	p-value	F-value	df	p-value
Short-term memory	-.24 (1.06)	-.61 (6)	.563	.56 (.81)	2.27 (10)	.046*	.26 (1.15)	1.22 (28)	.232	1.07 (1.06)	4.96 (23)	<.001***	3.924	3,67	.012*[a]
Working memory	-.66 (1.74)	-.93 (5)	.397	.95 (2.08)	1.52 (10)	.159	1.09 (1.76)	3.35 (28)	.002**	1.52 (2.17)	3.35 (22)	.003**	1.98	3,65	.126
Simple reaction time	-.47 (1.46)	-.86 (6)	.423	-.83 (1.47)	-1.87 (10)	.092	.06 (1.24)	.27 (28)	.792	.53 (1.63)	1.59 (23)	.127	2.548	3,67	.063
Selective attention	-.51 (1.03)	-1.32 (6)	.234	-.74 (.86)	-2.86 (10)	.017*	.31 (1.72)	.96 (28)	.344	.44 (1.95)	1.11 (23)	.278	1.759	3,67	.164
Inhibition	-1.27 (1.54)	-2.18 (6)	.072	-.31 (1.27)	-.80 (10)	.441	.37 (1.45)	1.36 (28)	.184	.63 (1.49)	2.02 (22)	.056	3.638	3,66	.017*[b]
Verbal memory	-.05 (1.20)	-.10 (6)	.921	.22 (1.08)	.67 (10)	.516	.73 (1.52)	2.59 (28)	.015*	.63 (1.54)	2.02 (23)	.056	.769	3,67	.516
Figural memory	-.52 (1.02)	-1.35 (6)	.225	.79 (1.29)	2.04 (10)	.069	.39 (1.30)	1.62 (28)	.117	.93 (1.11)	4.01 (22)	.001**	2.872	3,66	.043*[c]
Fluency	1.01 (1.14)	2.33 (6)	.059	.93 (.66)	4.72 (10)	.001**	.86 (1.40)	3.26 (27)	.003**	.69 (1.39)	2.39 (22)	.026*	.162	3,65	.922

Note.

Asterisks indicate statistically significant change of cognitive performance between T1 and T2 in *t*-test statistics, * $p < .05$, ** $p < .01$, *** $p < .001$

SD standard deviation, *df* degree of freedom

[#] ANOVAs (with F-statistics) refer to interaction effects (i.e. differences in extent of cognitive change between treatment groups: timepoint [T1 vs. T2] with group [RT vs. ChT vs. RChT vs. watchful-waiting]); asterisks indicate a statistically significant interaction effect

[a] Post-hoc t-test (with Bonferroni correction) indicates statistically significant group differences in extent of change between RT and watchful-waiting patients ($p = .032$) and between RChT and watchful-waiting ($p = .045$)

[b] Post-hoc t-test (with Bonferroni correction) indicates a statistically significant group difference in extent of change between RT and watchful-waiting patients ($p = .021$)

[c] Post-hoc t-test (with Bonferroni correction) indicates a statistically significant group difference in extent of change between RT and watchful-waiting patients ($p = .044$)
