# Supplementary material for: Long-term neurocognitive function and quality of life after multimodal therapy in adult glioma patients: a prospective long-term follow-up
Source: J Neurooncol. 2023 Aug 30;164(2):353–66. doi: 10.1007/s11060-023-04419-y (PMC10522752; doi:10.1007/s11060-023-04419-y)
Supplement: Supplementary file 3 — Supplementary file3 (PDF 89 KB) [file 11060_2023_4419_MOESM3_ESM.pdf]

Online Resource for the manuscript entitled: Long-term neurocognitive function and quality of life after multimodal therapy in adult glioma patients: A prospective long-term follow-up

Milena Pertz, Sabine Schlömer, Clemens Seidel, Bettina Hentschel, Markus Löffler, Gabriele Schackert, Dietmar Krex, Tareq Juratli, Joerg Christian Tonn, Oliver Schnell, Hartmut Vatter, Matthias Simon, Manfred Westphal, Tobias Martens, Michael Sabel, Martin Bendszus, Nils Dörner, Antje Wick, Klaus Fliessbach, Christian Hoppe, Marcel Klingner, Jörg Felsberg, Guido Reifenberger, Dorothee Gramatzki, Michael Weller, Uwe Schlegel for the German Glioma Network

Corresponding author: Milena Pertz

E-Mail address: milena.pertz@rub.de

Department of Medical Psychology and Medical Sociology, Ruhr University Bochum

Universitätsstraße 105, D-44789 Bochum, Germany

Journal name: Journal of Neuro-Oncology

Online Resource Table S3 Number of patients with a z-score ≥ 1.5 SD below the normative mean in cognitive domains, separated for treatment groups and RT dosage groups.

	**Whole sample (n=71)**				**Sample with RT plans available (n=27)**[#]	
	**Radiotherapy** (RT) n=7	**Combined Radio-Chemotherapy** (RChT) n=29	**Chemotherapy** (ChT) n=11	**Watchful-waiting** n=24	**Dmean ipsilateral Hippocampus <10 Gy** n=8	**Dmean ipsilateral Hippocampus >50 Gy** n=12
**Short-term memory**	T1: n = 0<br>T2: n = 1 (14.3%)	T1: n = 7 (25.0%)<br>T2: n = 4 (13.8%)	T1: n = 3 (27.3%)<br>T2: n = 2 (18.2%)	T1: n = 8 (33.3%)<br>T2: n = 3 (12.5%)	T1: n = 0<br>T2: n = 0	T1: n = 2 (16.7%)<br>T2: n = 3 (25.0%)
**Working memory**	T1: n = 0<br>T2: n = 1 (14.3%)	T1: n = 3 (10.3%)<br>T2: n = 2 (6.9%)	T1: n = 2 (18.2%)<br>T2: n = 1 (9.1%)	T1: n = 5 (20.8%)<br>T2: n = 1 (4.2%)	T1: n = 0<br>T2: n = 1 (12.5%)	T1: n = 2 (16.7%)<br>T2: n = 1 (8.3%)
**Simple reaction time**	T1: n = 1 (14.3%)<br>T2: n = 2 (28.6%)	T1: n = 6 (20.7%)<br>T2: n = 5 (17.2%)	T1: n = 0<br>T2: n = 2 (18.2%)	T1: n = 9 (37.5%)<br>T2: n = 4 (16.7%)	T1: n = 1 (12.5%)<br>T2: n = 1 (12.5%)	T1: n = 3 (25.0%)<br>T2: n = 4 (33.3%)
**Selective attention**	T1: n = 2 (28.6%)<br>T2: n = 2 (28.6%)	T1: n = 7 (25.0%)<br>T2: n = 4 (13.8%)	T1: n = 1 (9.1%)<br>T2: n = 3 (27.3%)	T1: n = 8 (33.3%)<br>T2: n = 4 (16.7%)	T1: n = 3 (37.5%)<br>T2: n = 1 (12.5%)	T1: n = 3 (25.0%)<br>T2: n = 3 (25.0%)
**Inhibition**	T1: n = 0<br>T2: n = 2 (28.6%)	T1: n = 6 (20.7%)<br>T2: n = 2 (6.9%)	T1: n = 1 (9.1%)<br>T2: n = 1 (9.1%)	T1: n = 8 (33.3%)<br>T2: n = 4 (16.7%)	T1: n = 3 (37.5%)<br>T2: n = 0	T1: n = 1 (8.3%)<br>T2: n = 2 (16.7%)
**Verbal memory**	T1: n = 2 (28.6%)<br>T2: n = 3 (42.9%)	T1: n = 13 (46.4%)<br>T2: n = 7 (25.0%)	T1: n = 2 (18.2%)<br>T2: n = 0	T1: n = 9 (37.5%)<br>T2: n = 4 (16.7%)	T1: n = 1 (12.5%)<br>T2: n = 0	T1: n = 7 (58.3%)<br>T2: n = 5 (41.7%)
**Figural memory**	T1: n = 1 (14.3%)<br>T2: n = 2 (28.6%)	T1: n = 5 (17.2%)<br>T2: n = 0	T1: n = 1 (9.1%)<br>T2: n = 1 (9.1%)	T1: n = 6 (25.0%)<br>T2: n = 1 (4.2%)	T1: n = 0<br>T2: n = 0	T1: n = 3 (25.0%)<br>T2: n = 0
**Fluency**	T1: n = 2 (28.6%)<br>T2: n = 1 (14.3%)	T1: n = 5 (17.2%)<br>T2: n = 1 (3.4%)	T1: n = 2 (18.2%)<br>T2: n = 0	T1: n = 5 (20.8%)<br>T2: n = 2 (8.3%)	T1: n = 2 (25.0%)<br>T2: n = 0	T1: n = 2 (16.7%)<br>T2: n = 1 (8.3%)

Note. *T1* baseline neuropsychological assessment, *T2* follow-up neuropsychological assessment
[#] for n=6 patients hippocampal dosage was between 10 Gy and 50 Gy; for n=1 patient estimation of hippocampal dosage was not possible; data of these patients were not included in one of predefined dichotomized groups
