# Supplementary material for: Long-term neurocognitive function and quality of life after multimodal therapy in adult glioma patients: a prospective long-term follow-up
Source: J Neurooncol. 2023 Aug 30;164(2):353–66. doi: 10.1007/s11060-023-04419-y (PMC10522752; doi:10.1007/s11060-023-04419-y)
Supplement: Supplementary file 4 — Supplementary file4 (PDF 36 KB) [file 11060_2023_4419_MOESM4_ESM.pdf]

Online Resource for the manuscript entitled: Long-term neurocognitive function and quality of life after multimodal therapy in adult glioma patients: A prospective long-term follow-up

Milena Pertz, Sabine Schlömer, Clemens Seidel, Bettina Hentschel, Markus Löffler, Gabriele Schackert, Dietmar Krex, Tareq Juratli, Joerg Christian Tonn, Oliver Schnell, Hartmut Vatter, Matthias Simon, Manfred Westphal, Tobias Martens, Michael Sabel, Martin Bendszus, Nils Dörner, Antje Wick, Klaus Fliessbach, Christian Hoppe, Marcel Klingner, Jörg Felsberg, Guido Reifenberger, Dorothee Gramatzki, Michael Weller, Uwe Schlegel for the German Glioma Network

Corresponding author: Milena Pertz

E-Mail address: milena.pertz@rub.de

Department of Medical Psychology and Medical Sociology, Ruhr University Bochum

Universitätsstraße 105, D-44789 Bochum, Germany

Journal name: Journal of Neuro-Oncology

Online Resource Table S4 Comparison of individual test scores of patients with AED at T1 and no AED at T2 (n = 7) to group mean scores of respective subtests, shown separately for treatment groups (patient 1-7) and for hippocampal dosimetry (patients 2, 4).

		**Individual test scores with a greater extent of neurocognitive improvement relative to group mean scores**								Number of subtests with a greater extent of improvement in individual test scores
**Patient**	**Treatment group**	**Short-term memory**	**Working memory**	**Simple reaction time**	**Selective attention**	**Inhibition**	**Verbal memory**	**Figural memory**	**Fluency**	
1	RChT (n=19)	n.s.	n.s.	n.s.	n.s.	n.s.	n.s.	n.s.	n.s.	0/8
2	RChT (n=19)	n.s.	n.s.	n.s.	n.s.	n.s.	n.s.	n.s.	n.s.	0/8
3	RChT (n=19)	*t* = -3.174; *p* = .005	*t* = -5.911; *p* < .001	n.s.	n.s.	n.s.	*t* = -3.688; *p* = .002	n.s.	n.s.	3/8
4	RChT (n=19)	n.s.	n.s.	n.s.	*t* = -7.906; *p* < .001	*t* = -11.818; *p* < .001	n.s.	n.s.	n.s.	2/8
5	ChT (n=9)	n.s.	n.s.	n.s.	n.s.	n.s.	n.s.	n.s.	n.s.	0/8
6	ChT (n=9)	n.s.	*t* = -4.274; *p* = .003	n.s.	*t* = -2.516; *p* = .036	n.s.	n.s.	n.s.	n.s.	2/8
7	WW (n=22)	n.s.	*t* = -2.759; *p* = .012	n.s.	*t* = -3.184; *p* = .004	n.s.	*t* = -5.616; *p* < .001	n.s.	n.s.	3/8
4	< 10 Gy (n=4)	n.s.	n.s.	n.s.	*t* = -8.357; *p* = .004	*t* = -5.560; *p* = .011	n.s.	n.s.	n.s.	2/8
2	> 50 Gy (n=10)	n.s.	n.s.	n.s.	n.s.	n.s.	n.s.	n.s.	n.s.	0/8

Note. *AED* anti-epileptic drug, *RChT* combined radio-chemotherapy, *ChT* chemotherapy, *WW* watchful-waiting, *n.s.* not significant

Each individual performance score was compared to group mean scores of respective subtests. For calculation of group mean scores, individual scores of patients who received AED at T1 but no AED at T2 (n=7) or who received AED at T1 but had lacking AED information at T2 (n=7) were excluded. Results of one-sample *t*-tests are shown.
