# Supplementary material for: Long-term neurocognitive function and quality of life after multimodal therapy in adult glioma patients: a prospective long-term follow-up
Source: J Neurooncol. 2023 Aug 30;164(2):353–66. doi: 10.1007/s11060-023-04419-y (PMC10522752; doi:10.1007/s11060-023-04419-y)
Supplement: Supplementary file 5 — Supplementary file5 (PDF 292 KB) [file 11060_2023_4419_MOESM5_ESM.pdf]

Online Resource for the manuscript entitled: Long-term neurocognitive function and quality of life after multimodal therapy in adult glioma patients: A prospective long-term follow-up

Milena Pertz, Sabine Schlömer, Clemens Seidel, Bettina Hentschel, Markus Löffler, Gabriele Schackert, Dietmar Krex, Tareq Juratli, Joerg Christian Tonn, Oliver Schnell, Hartmut Vatter, Matthias Simon, Manfred Westphal, Tobias Martens, Michael Sabel, Martin Bendszus, Nils Dörner, Antje Wick, Klaus Fliessbach, Christian Hoppe, Marcel Klingner, Jörg Felsberg, Guido Reifenberger, Dorothee Gramatzki, Michael Weller, Uwe Schlegel for the German Glioma Network

Corresponding author: Milena Pertz

E-Mail address: milena.pertz@rub.de

Department of Medical Psychology and Medical Sociology, Ruhr University Bochum

Universitätsstraße 105, D-44789 Bochum, Germany

Journal name: Journal of Neuro-Oncology

Online Resource Figure S5 Mean values and standard deviations (error bars) of EORTC QLQ C30 functional scales and Global Health Status at baseline and follow-up (median 4.6 years [range 1.4-9.0] after baseline), separated for treatment groups. a) Watchful-waiting (n = 24), b) Chemotherapy (n = 11), c) Radiotherapy (n = 7), d) Combined radio-chemotherapy (n = 29). Statistically significant changes in quality of life between baseline and follow-up are indicated by asterisks (* $p < .05$).

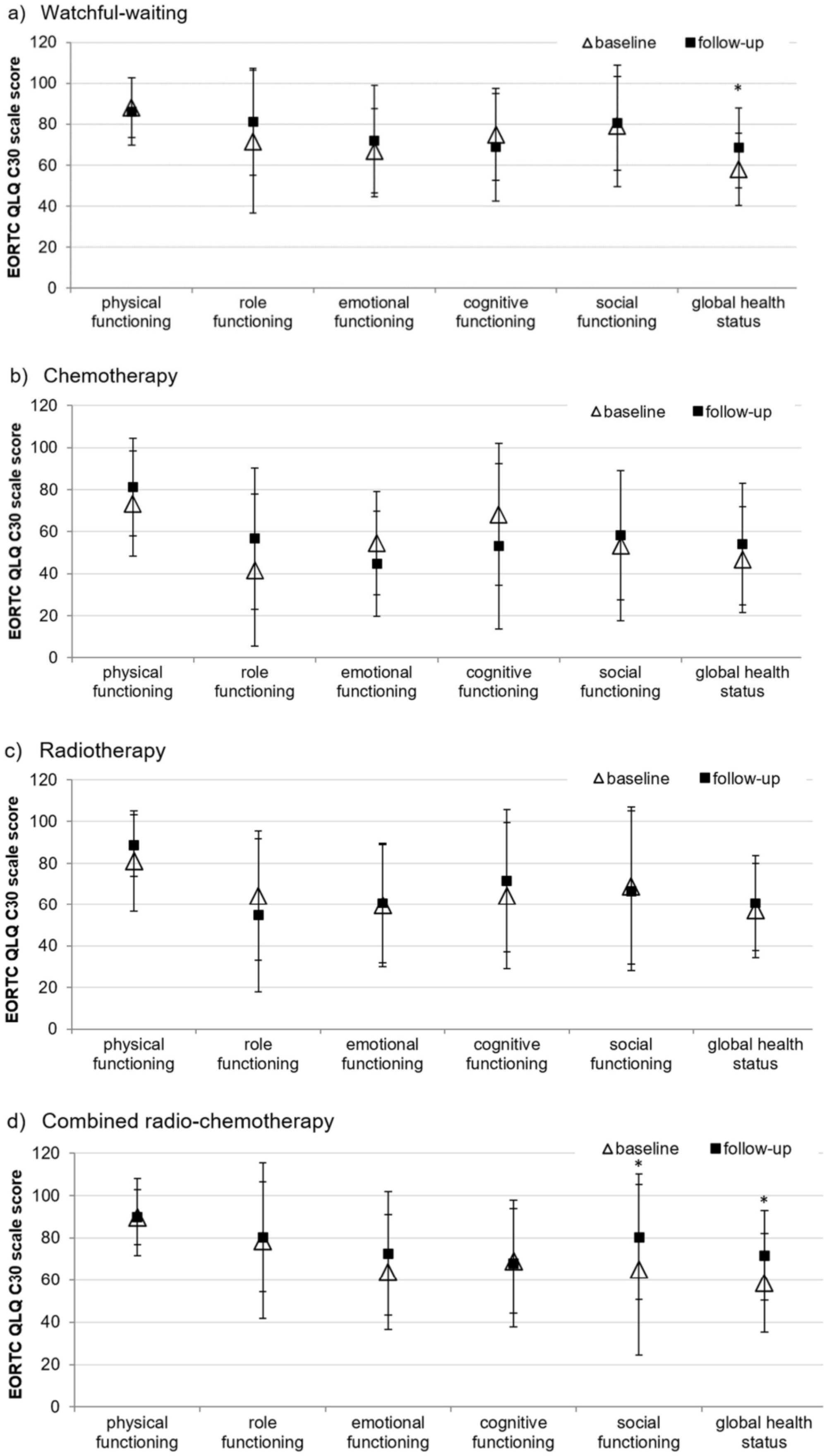
a) Watchful-waiting
b) Chemotherapy
c) Radiotherapy
d) Combined radio-chemotherapy
EORTC QLQ C30 scale score
Δ baseline
■ follow-up
physical functioning
role functioning
emotional functioning
cognitive functioning
social functioning
global health status
0
20
40
60
80
100
120
*
