# Supplementary material for: Long-term neurocognitive function and quality of life after multimodal therapy in adult glioma patients: a prospective long-term follow-up
Source: J Neurooncol. 2023 Aug 30;164(2):353–66. doi: 10.1007/s11060-023-04419-y (PMC10522752; doi:10.1007/s11060-023-04419-y)
Supplement: Supplementary file 6 — Supplementary file6 (PDF 24 KB) [file 11060_2023_4419_MOESM6_ESM.pdf]

Online Resource for the manuscript entitled: Long-term neurocognitive function and quality of life after multimodal therapy in adult glioma patients: A prospective long-term follow-up

Milena Pertz, Sabine Schlömer, Clemens Seidel, Bettina Hentschel, Markus Löffler, Gabriele Schackert, Dietmar Krex, Tareq Juratli, Joerg Christian Tonn, Oliver Schnell, Hartmut Vatter, Matthias Simon, Manfred Westphal, Tobias Martens, Michael Sabel, Martin Bendszus, Nils Dörner, Antje Wick, Klaus Fliessbach, Christian Hoppe, Marcel Klingner, Jörg Felsberg, Guido Reifenberger, Dorothee Gramatzki, Michael Weller, Uwe Schlegel for the German Glioma Network

Corresponding author: Milena Pertz

E-Mail address: milena.pertz@rub.de

Department of Medical Psychology and Medical Sociology, Ruhr University Bochum

Universitätsstraße 105, D-44789 Bochum, Germany

Journal name: Journal of Neuro-Oncology

Online Resource Table S6 Cognitive performance in group means (M) and standard deviations (SD) for baseline (T1) and follow-up (T2) neuropsychological assessment (in z-scores), separated for patients with Dmean ipsilateral Hippocampus < 10 Gy and > 50 Gy.

	**Dmean ipsilateral Hippocampus < 10 Gy (n = 8)**		**Dmean ipsilateral Hippocampus > 50 Gy (n = 12)**		**ANOVA** [#]		
	M_{T1} (SD)	M_{T2} (SD)	M_{T1} (SD)	M_{T2} (SD)	F-value	df	*p*-value
Short-term memory (digit span)	-.22 (.70)	.14 (1.08)	-.36 (1.13)	-.66 (1.15)	1.186	1,18	.291
Working memory (two back)	-.09 (1.36)	1.41 (1.98)	.19 (1.82)	.69 (2.04)	.964	1,18	.339
Simple reaction time (alertness)	-.47 (1.24)	-.22 (1.15)	-.69 (1.44)	-1.18 (1.40)	1.582	1,18	.225
Selective attention (go/nogo)	-1.12 (1.46)	-.48 (1.0)	-.80 (1.37)	-1.06 (1.47)	1.298	1,18	.269
Inhibition (go/nogo)	-1.09 (1.79)	.14 (.74)	-.10 (1.0)	-.73 (1.30)	6.472	1,18	.020*[a]
Verbal memory	-.34 (1.29)	.72 (.94)	-1.26 (1.75)	-.92 (1.37)	.891	1,18	.358
Figural memory	-.03 (.43)	.09 (.98)	-.49 (1.36)	-.01 (.84)	.404	1,18	.533
Fluency	-.64 (1.25)	.71 (1.30)	-.33 (1.59)	.12 (1.25)	3.713	1,18	.070

Note.
df degree of freedom, * *p* < .05
[#] ANOVAs (with F-statistics) refer to interaction effects (i.e. differences in cognitive change between groups: timepoint [T1 vs. T2] with group [Dmean ipsilateral Hippocampus < 10 Gy vs. > 50 Gy]); the asterisk indicates a statistically significant interaction effect. For n=6 patients hippocampal dose was between 10 Gy and 50 Gy; for n=1 patient estimation of hippocampal dose was not possible; data of these patients were not included in the analyses of predefined dichotomized groups
[a] Patients with Dmean < 10 Gy show a statistically stronger change in the NeuroCog FX subtest *inhibition* between T1 and T2 (i.e. an improvement) than patients with Dmean > 50 Gy (who show a deterioration)
