# Supplementary material for: Long-term neurocognitive function and quality of life after multimodal therapy in adult glioma patients: a prospective long-term follow-up
Source: J Neurooncol. 2023 Aug 30;164(2):353–66. doi: 10.1007/s11060-023-04419-y (PMC10522752; doi:10.1007/s11060-023-04419-y)

Online Resource for the manuscript entitled: Long-term neurocognitive function and quality of life after multimodal therapy in adult glioma patients: A prospective long-term follow-up

Milena Pertz, Sabine Schlömer, Clemens Seidel, Bettina Hentschel, Markus Löffler, Gabriele Schackert, Dietmar Krex, Tareq Juratli, Joerg Christian Tonn, Oliver Schnell, Hartmut Vatter, Matthias Simon, Manfred Westphal, Tobias Martens, Michael Sabel, Martin Bendszus, Nils Dörner, Antje Wick, Klaus Fliessbach, Christian Hoppe, Marcel Klingner, Jörg Felsberg, Guido Reifenberger, Dorothee Gramatzki, Michael Weller, Uwe Schlegel for the German Glioma Network

Corresponding author: Milena Pertz

E-Mail address: milena.pertz@rub.de

Department of Medical Psychology and Medical Sociology, Ruhr University Bochum

Universitätsstraße 105, D-44789 Bochum, Germany

Journal name: Journal of Neuro-Oncology

Online Resource Figure S7 Mean EORTC-QLQ C30 functional scale scores and Global Health Status in relation to hippocampal dosage. Graphs indicate mean scale scores in the long-term on group level with standard deviations represented by error bars at baseline (i.e. after surgery) and at follow-up (median 4.6 years [range 1.4-9.0] after baseline), separated for patients with mean ipsilateral hippocampal dosage < 10 Gy (n = 8) and patients with mean ipsilateral hippocampal dosage > 50 Gy (n = 12).

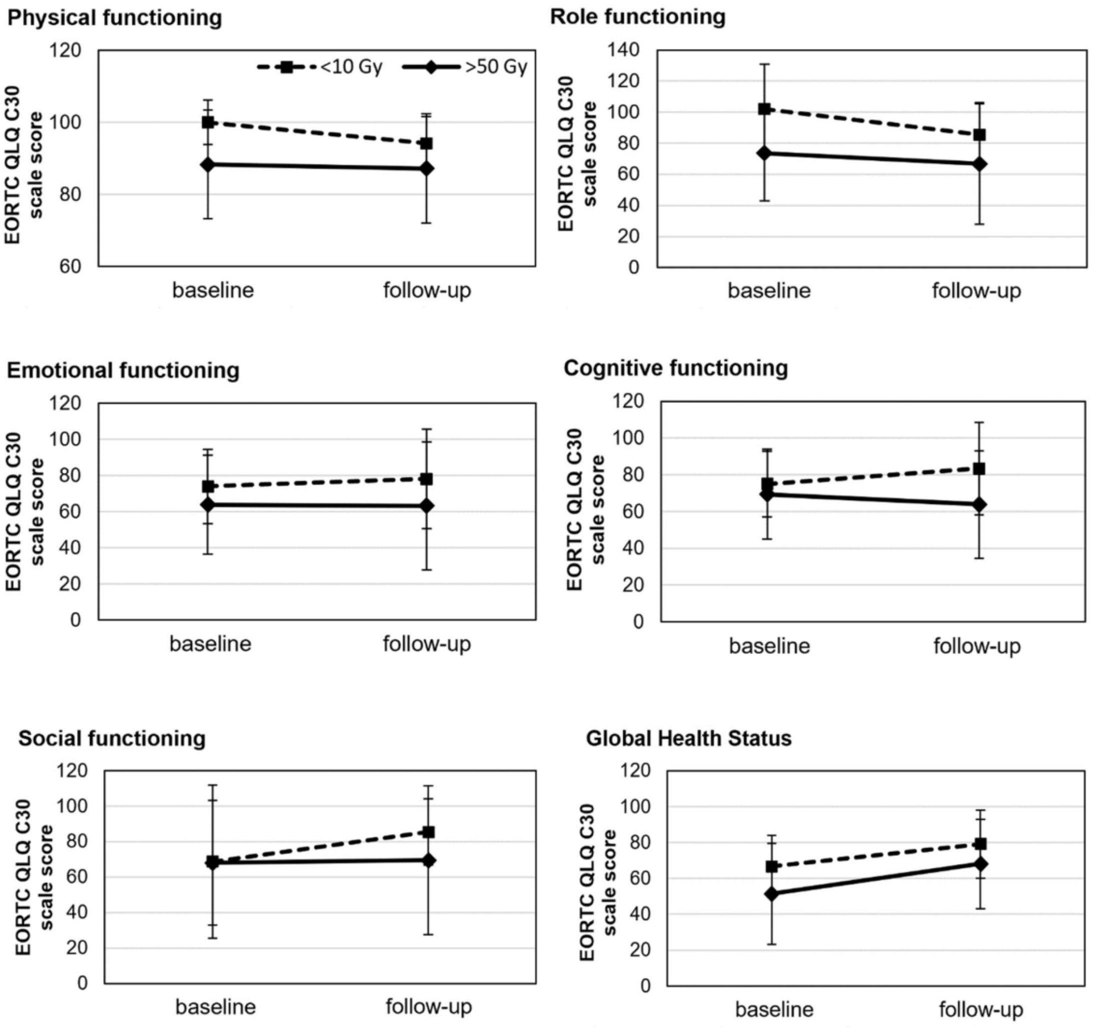

Supplement: Supplementary file 7 — Supplementary file7 (PDF 265 KB) [file 11060_2023_4419_MOESM7_ESM.pdf]
