# Supplementary material for: Long-term neurocognitive function and quality of life after multimodal therapy in adult glioma patients: a prospective long-term follow-up
Source: J Neurooncol. 2023 Aug 30;164(2):353–66. doi: 10.1007/s11060-023-04419-y (PMC10522752; doi:10.1007/s11060-023-04419-y)
Supplement: Supplementary file 8 — Supplementary file8 (PDF 21 KB) [file 11060_2023_4419_MOESM8_ESM.pdf]

Online Resource for the manuscript entitled: Long-term neurocognitive function and quality of life after multimodal therapy in adult glioma patients: A prospective long-term follow-up

Milena Pertz, Sabine Schlömer, Clemens Seidel, Bettina Hentschel, Markus Löffler, Gabriele Schackert, Dietmar Krex, Tareq Juratli, Joerg Christian Tonn, Oliver Schnell, Hartmut Vatter, Matthias Simon, Manfred Westphal, Tobias Martens, Michael Sabel, Martin Bendszus, Nils Dörner, Antje Wick, Klaus Fliessbach, Christian Hoppe, Marcel Klingner, Jörg Felsberg, Guido Reifenberger, Dorothee Gramatzki, Michael Weller, Uwe Schlegel for the German Glioma Network

Corresponding author: Milena Pertz

E-Mail address: milena.pertz@rub.de

Department of Medical Psychology and Medical Sociology, Ruhr University Bochum

Universitätsstraße 105, D-44789 Bochum, Germany

Journal name: Journal of Neuro-Oncology

Online Resource Table S8 Quality of life in group means (M) and standard deviations (SD) for baseline (T1) and follow-up (T2), separated for patients with Dmean ipsilateral Hippocampus < 10 Gy vs. > 50 Gy.

	**Dmean ipsilateral Hippocampus < 10 Gy (n = 8)**		**Dmean ipsilateral Hippocampus > 50 Gy (n = 12)**		**ANOVA #**		
	M_{T1} (SD)	M_{T2} (SD)	M_{T1} (SD)	M_{T2} (SD)	F-value	df	*p*-value
Physical functioning	100.0 (6.17)	94.17 (7.51)	88.33 (15.08)	87.22 (15.16)	.857	1,18	.367
Role functioning	102.08 (28.78)	85.42 (20.77)	73.61 (30.53)	66.67 (38.92)	.291	1,18	.596
Emotional functioning	73.96 (20.62)	78.13 (27.44)	63.89 (27.37)	63.20 (35.44)	.112	1,18	.742
Cognitive functioning	75.0 (17.82)	83.33 (25.20)	69.44 (24.45)	63.89 (29.16)	1.406	1,18	.251
Social functioning	68.75 (43.13)	85.42 (18.77)	68.06 (35.15)	69.45 (41.94)	.538	1,18	.473
Global Health Status	66.67 (17.25)	79.17 (18.90)	51.39 (28.17)	68.06 (24.83)	.072	1,18	.792

Note.
df degree of freedom
ANOVAs (with F-statistics) refer to interaction effects (i.e. differences in change of QoL between groups: timepoint [T1 vs. T2] with group [Dmean ipsilateral Hippocampus < 10 Gy vs. > 50 Gy]). For n=6 patients hippocampal dose was between 10 Gy and 50 Gy; for n=1 patient estimation of hippocampal dose was not possible; data of these patients were not included in the analyses of predefined dichotomized groups
