# Supplementary material for: Long-term neurocognitive function and quality of life after multimodal therapy in adult glioma patients: a prospective long-term follow-up
Source: J Neurooncol. 2023 Aug 30;164(2):353–66. doi: 10.1007/s11060-023-04419-y (PMC10522752; doi:10.1007/s11060-023-04419-y)
Supplement: Supplementary file 9 — Supplementary file9 (PDF 353 KB) [file 11060_2023_4419_MOESM9_ESM.pdf]

Online Resource for the manuscript entitled: Long-term neurocognitive function and quality of life after multimodal therapy in adult glioma patients: A prospective long-term follow-up

Milena Pertz, Sabine Schlömer, Clemens Seidel, Bettina Hentschel, Markus Löffler, Gabriele Schackert, Dietmar Krex, Tareq Juratli, Joerg Christian Tonn, Oliver Schnell, Hartmut Vatter, Matthias Simon, Manfred Westphal, Tobias Martens, Michael Sabel, Martin Bendszus, Nils Dörner, Antje Wick, Klaus Fliessbach, Christian Hoppe, Marcel Klingner, Jörg Felsberg, Guido Reifenberger, Dorothee Gramatzki, Michael Weller, Uwe Schlegel for the German Glioma Network

Corresponding author: Milena Pertz

E-Mail address: milena.pertz@rub.de

Department of Medical Psychology and Medical Sociology, Ruhr University Bochum

Universitätsstraße 105, D-44789 Bochum, Germany

Journal name: Journal of Neuro-Oncology

Online Resource Figure S9 Visualization of correlation analyses of cognitive change (T2 – T1 in z-scores) in NeuroCog FX subtests and size of planning target volume (PTV). Each circle represents a patient with available RT plans and available PTV size, n = 26.

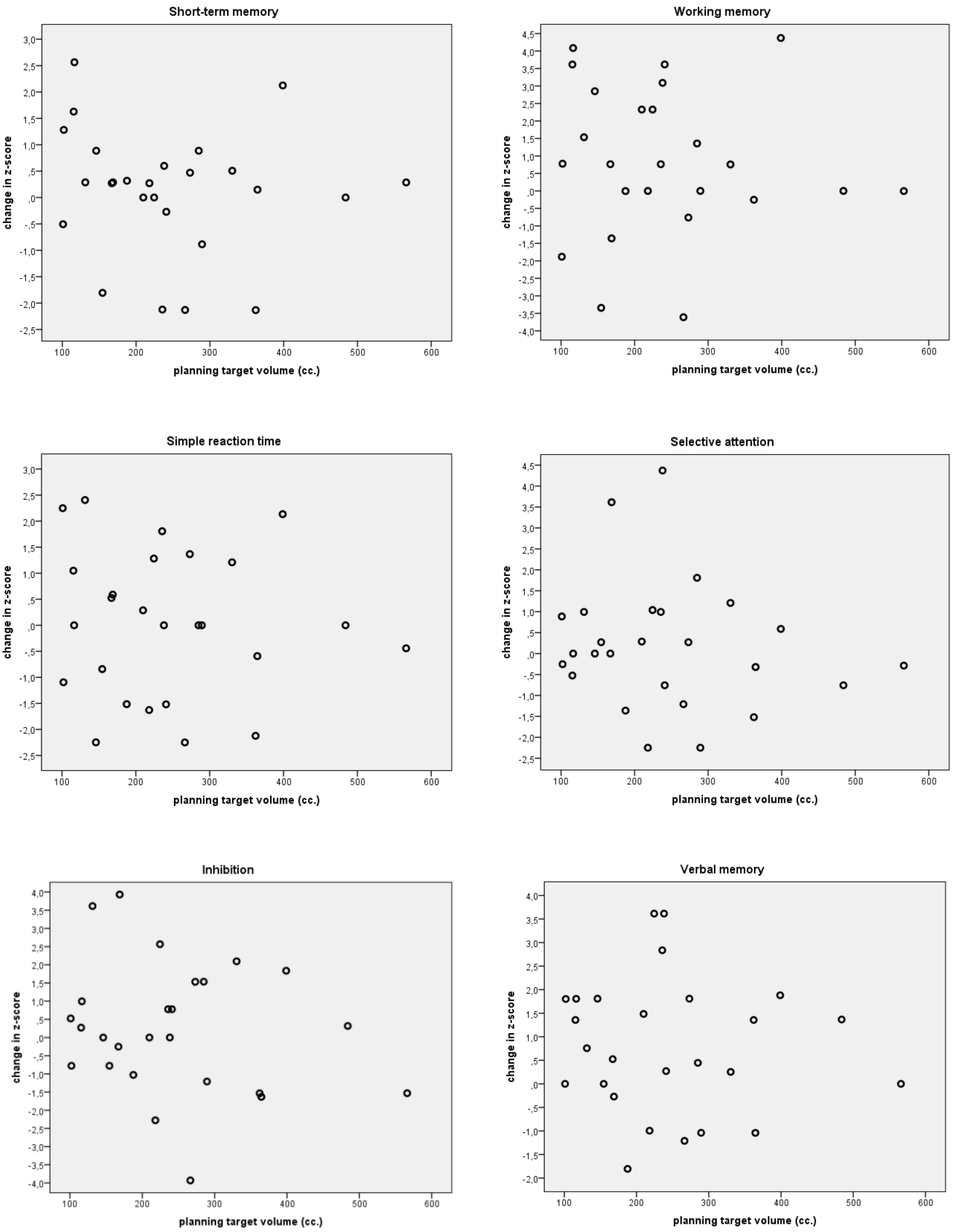

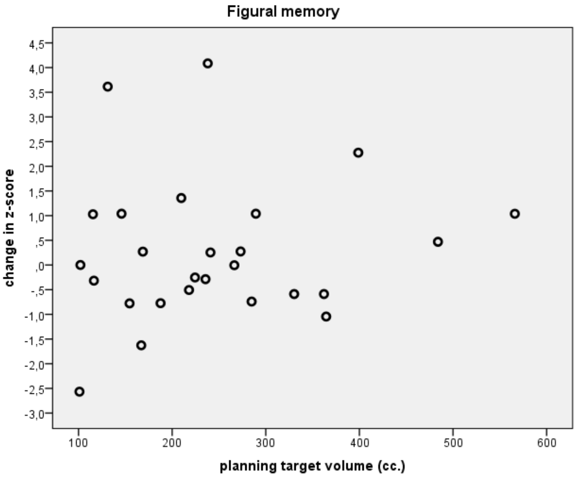
Figural memory
change in z-score
planning target volume (cc.)

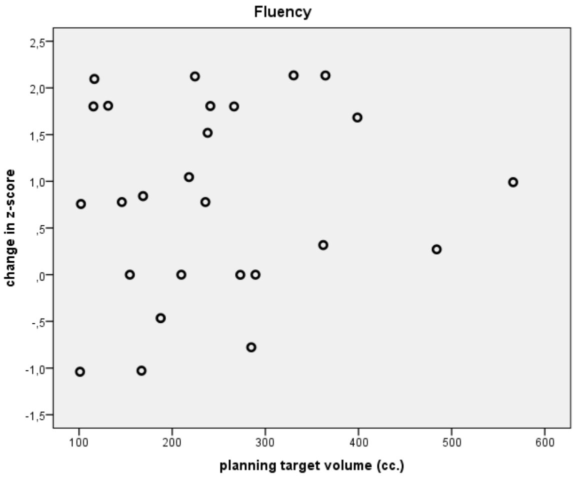
Fluency
change in z-score
planning target volume (cc.)

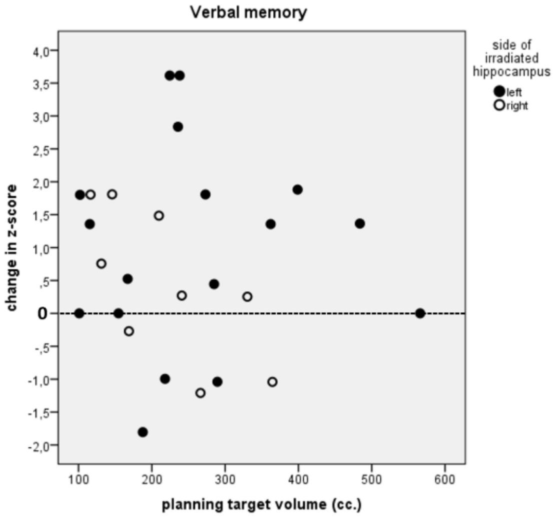
Verbal memory
change in z-score
planning target volume (cc.)
side of irradiated hippocampus
left
right

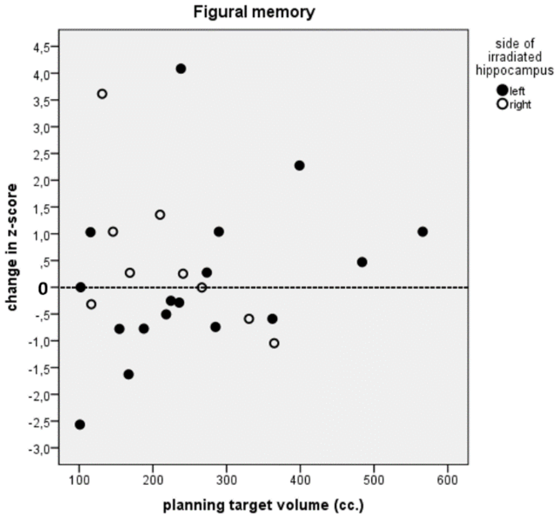
Figural memory
change in z-score
planning target volume (cc.)
side of irradiated hippocampus
left
right
